# T-FINDER: A highly sensitive, pan-HLA platform for functional T cell receptor and ligand discovery

**DOI:** 10.1126/sciadv.adk3060

**Published:** 2024-02-02

**Authors:** Miray Cetin, Veronica Pinamonti, Theresa Schmid, Tamara Boschert, Ana Mellado Fuentes, Kristina Kromer, Taga Lerner, Jing Zhang, Yonatan Herzig, Christopher Ehlert, Miguel Hernandez-Hernandez, Georgios Samaras, Claudia Maldonado Torres, Laura Fisch, Valeriia Dragan, Arlette Kouwenhoven, Bertrand Van Schoubroeck, Hans Wils, Carl Van Hove, Michael Platten, Edward W. Green, Frederik Stevenaert, Nathan J. Felix, John M. Lindner

**Affiliations:** ^1^ BioMed X GmbH, Im Neuenheimer Feld 515, 69120 Heidelberg, Germany.; ^2^ Faculty of Biosciences, Heidelberg University, 69120 Heidelberg, Germany.; ^3^ DKTK CCU Neuroimmunology and Brain Tumor Immunology, German Cancer Research Center (DKFZ), 69120 Heidelberg, Germany.; ^4^ Helmoltz Institute for Translational Oncology (HI-TRON), Heidelberg, Germany.; ^5^ Heidelberg Institute for Theoretical Studies (HITS gGmbH), 69118 Heidelberg, Germany.; ^6^ Janssen Research and Development, Beerse, Belgium.; ^7^ Department of Neurology, Medical Faculty Mannheim, MCTN Heidelberg University, Mannheim, Germany.; ^8^ Janssen Research and Development, Spring House, PA, USA.

## Abstract

Effective, unbiased, high-throughput methods to functionally identify both class II and class I HLA–presented T cell epitopes and their cognate T cell receptors (TCRs) are essential for and prerequisite to diagnostic and therapeutic applications, yet remain underdeveloped. Here, we present T-FINDER [T cell Functional Identification and (Neo)-antigen Discovery of Epitopes and Receptors], a system to rapidly deconvolute CD4 and CD8 TCRs and targets physiologically processed and presented by an individual’s unmanipulated, complete human leukocyte antigen (HLA) haplotype. Combining a highly sensitive TCR signaling reporter with an antigen processing system to overcome previously undescribed limitations to target expression, T-FINDER both robustly identifies unknown peptide:HLA ligands from antigen libraries and rapidly screens and functionally validates the specificity of large TCR libraries against known or predicted targets. To demonstrate its capabilities, we apply the platform to multiple TCR-based applications, including diffuse midline glioma, celiac disease, and rheumatoid arthritis, providing unique biological insights and showcasing T-FINDER’s potency and versatility.

## Teaser

The modular T-FINDER platform enables rapid and robust characterization of disease-relevant T cell receptors and ligands.

## INTRODUCTION

The molecular interaction between T cell receptors (TCRs) and their cognate ligands instructs both functions and dysfunctions of the adaptive immune system. T cells express clonally unique TCRs, and next-generation sequencing can provide cohorts of “TCRs of interest” based on phenotype and/or extent of clonal expansion (*1*), but no information regarding functional specificity. Mapping cognate ligands of disease-relevant TCRs provides insights into etiopathology and guides therapeutic strategies; however, this remains a technologically challenging goal. Existing TCR deconvolution strategies include cell-based reporter systems, which are predominantly applied to CD8 TCRs and class I human leukocyte antigen (HLA) complex-presented antigens (*2*, *3*). Several more recently established approaches address CD4 TCR deconvolution (*4*, *5*), but their general utility, scalability, and capacity for de novo epitope discovery remain unclear. In silico approaches predicting the identity and HLA restriction of a TCR ligand, while offering promising throughput and efficiency, are limited by the quality of their training datasets, and generalize poorly to epitopes that do not share structural similarity to the training data (*6*, *7*). Major obstacles to deconvoluting TCR specificities include HLA diversity (*8*) (more than 12,000 known alleles) and the ability to screen a proteome-wide library simultaneously for class II– and class I–presented epitopes.

To functionally identify TCR ligands, we designed a highly sensitive TCR deconvolution platform that avoids engineering the molecular TCR:target interaction, is agnostic to predicted epitopes and presenting alleles, operates for both class II and class I HLA–presented peptides, and is independent from mass spectrometry, tetramer labeling, and other ligand/TCR prediction steps. Particularly, it is unclear to what extent antigen-presenting cells (APCs) can process and present epitopes derived from intracellularly expressed targets on class II HLA complexes. These hurdles necessitated the development of several unique tools, including greatly improving the sensitivity of TCR-inducible promoters and reporters and establishing a method to achieve potent class II antigen processing and presentation. After establishing and benchmarking our platform, we performed functional ligand deconvolution screens across infectious disease, autoimmunity, and oncology, demonstrating the flexibility and potency of the system. On the basis of its ability to physiologically identify and intrinsically validate TCRs and their epitopes, we named this platform T-FINDER [T cell Functional Identification and (Neo)-antigen Discovery of Epitopes and Receptors].

## RESULTS

### Generating a TCR reporter system with high sensitivity and signal-to-noise ratio

**Fig. 1.**
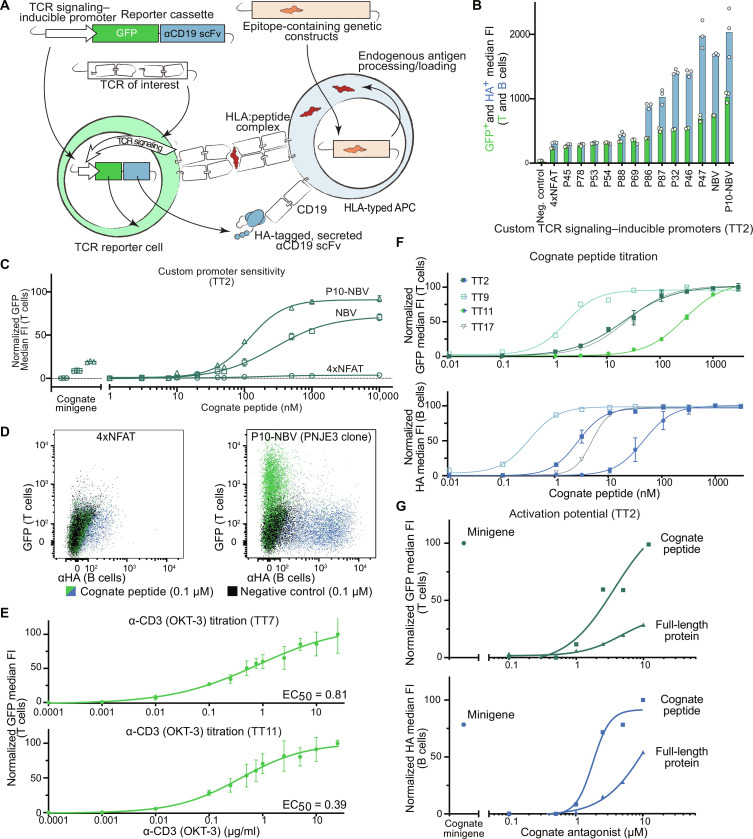
Developing and benchmarking a TCR activation reporter system. (**A**) Schematic depicting construction of a TCR reporter cell line (left) transgenically modified with an inducible promoter and TCR of interest. Upon cognate ligand interaction, the reporter cell produces cytoplasmic GFP and an HA-labeled secreted antibody fragment specific for CD19, a B cell-specific surface protein. B cells (right) used as APCs are transduced with epitope containing minigenes or exogenously loaded with peptide epitopes. Upon reporter T cell activation, the B cells are labeled by the secreted αCD19 scFv. (**B**) Median fluorescent GFP (green) and αHA (blue) signals upon cognate ligand interaction for TT2-transgenic Jurkat cells with bespoke TCR signaling–inducible promoters. Negative control, noncognate interaction (from P10-NBV promoter as an example). (**C**) Minigene and peptide dilution curves of a cognate epitope for P10-NBV, NBV, and 4xNFAT transposon-integrated inducible reporter cassettes (bulk T cell population before single-clone selection). (**D**) Superimposed dot plots depicting T cell GFP expression (*y* axis, green) and B cell anti-CD19 scFv labeling (*x* axis, blue) in a single cognate-target coculture. Black dots, GFP fluorescence in a noncognate control coculture. Left: 4xNFAT promoter. Right: P10-NBV promoter-transgenic single clone PNJE3. (**E**) TCR-independent titration curves for two benchmarking TCRs (TT7 and TT11) using an αCD3 antibody (OKT-3). (**F**) Titration curves of PNJE3 activation of four benchmarking TCRs (TT2, TT9, TT11, and TT17) with cognate peptide epitopes from the *Clostridium tetani* tetanospasmin protein. Upper plot, T cell GFP expression; lower plot, B cell anti-CD19 scFv labeling. Error bars indicate SD from three replicates. (**G**) Activation of PNJE3 in coculture with BOLETH cells following cognate peptide and full-length tetanus toxoid protein electropulsing, or transduction with an epitope-containing 400–amino acid minigene. Representative data from two independent titration experiments.

To maximize discriminatory potential and flexibility, we designed a TCR reporter with both T cell–intrinsic and T cell–extrinsic T cell activation readouts. Cytoplasmic production of green fluorescent protein (GFP) (*9*) provides T cell fluorescence easily detectable by flow cytometry or microscopy, while a secreted APC-specific single-chain variable fragment (scFv) labels nearby APCs for direct identification, e.g., by flow cytometry (Fig. 1A). This APC-labeling feature is particularly advantageous for high-throughput applications such as microfluidic emulsion nanoassays, in which APCs encoding the cognate epitope(s) must be isolated from the activated reporter cells (fig. S1A).

To combat weak signals from low-affinity/avidity interactions, we generated a highly sensitive synthetic TCR-inducible promoter over several iterations, benchmarking constructs using both published TCR:pHLA pairs (table S1) and internally generated tetanus toxoid (TT) reactive TCRs (TT2, TT9, TT7, and TT11 shown in Fig. 1). Established promoter elements [e.g., four tandem copies of the nuclear factor of activated T cell (NFAT) binding sequence] did not provide sufficient signal-to-noise sensitivity as lentiviral integrants (fig. S1, B and F) and were practically nonfunctional as transposon cassettes (Fig. 1, B and D, and fig. S1, C and D). Therefore, we designed a series of promoters (P1 to P15) using permutations of 60-bp genomic fragments from TCR response loci (*10*, *11*) centered around NFAT binding sites, as well as fully synthetic combinations of transcription factor binding sequences. For the latter, we included binding sites for nuclear factor κB, Fos/Jun (AP-1), and Oct1/2-Bob1 to maximally integrate TCR-inducible pathways. These synthetic designs also accounted for binding motif spacing to minimize steric hindrance and competition among transcriptional activators on the DNA (*12*). The “NBV” (NFAT-Bob1-Virtually designed) promoter yielded an approximately 10-fold signal-to-noise ratio when integrated via a lentiviral transgene, increasing to nearly 50-fold when using a genomically protected transposon (fig. S1C). A similarly constructed second series of promoters (P45-P90) provided building blocks for ‘tunable’ promoters with a range of signal strengths (Fig. 1B), which can be selected together with additional coculture parameters to provide relatively quantitative readouts for TCR activity within a given application. However, the greatest signal-to-noise ratio was achieved by combining the NBV promoter with the P10 genomic reassembly fragment (P10-NBV; Fig. 1, B and C; and fig. S1D).

Following single-cell cloning and selection of a P10-NBV reporter clone (Fig. 1D), we assessed its sensitivity to serial dilutions of anti-CD3 antibody (Fig. 1E and fig. S1E) and in vitro loaded peptides (Fig. 1F). While the reporter cassette itself is functional in T cell lines including Jurkat (Fig. 1, B to G) and SKW-3 (fig. S1F), the Jurkat-based clone PNJE3 demonstrated nanomolar EC_50_ values with linear response ranges over several orders of magnitude. Notably, coculturing 50 TCRs in the absence of cognate ligand, the T-FINDER readouts were cleaner than CD69 surface expression, a typical proximal indicator of TCR signaling–mediated activation, suggesting that while CD69 levels reflect antigen-independent, tonic signaling of many TCRs, PNJE3 cells are only activated in response to complete T cell engagement by cognate ligands (fig. S1G). In the PNJE3 reporter line, GFP greatly outperforms the activation-associated marker 4-1BB upon cognate interaction; when studying weak TCRs and/or poorly presented epitopes, the APC-labeling scFv fragment provides an additional advantage as a saturating secondary signal (fig. S1I).

### Enabling natural antigen processing of genetically encoded targets

PNJE3 cells are compatible with all types of epitope presentation, including tumor cell lines and engineered APCs (*13*). However, when performing de novo target identification for a given donor, simultaneously interrogating entire HLA haplotypes is most efficient. For this, we used Epstein-Barr virus–immortalized B lymphocytic cell lines (B-LCLs) as autologous APCs, observing they could competently process full-length tetanus toxoid protein into peptide epitopes (Fig. 1G). Notably, HLA zygosity did not after overall sensitivity; we obtained equivalent results using DR4-homozygous (BOLETH) (*14*) and hemizygous B-LCLs (fig. S1H), indicating that donor-derived B cell lines, which recapitulate the entire complement of HLA alleles, can be used to identify TCR:epitope pairs.

Moving beyond in vitro loaded peptides, we introduced genetically encoded antigens as 400–amino acid open reading frames (“minigenes”) linked via a T2A viral peptide (*15*) to mTagBFP for reading frame and expression validation (“2B” backbone). Minigenes may contain full-length proteins, overlapping fragments thereof (e.g., 30–amino acid tiling to avoid joints that may interrupt peptide epitopes (*16*)], or multiple proteins or fragments joined by flexible linkers (fig. S2A). This step multiplexes peptide epitopes and ensures that large antigen libraries can be screened at sufficient depth, which is not practical using, e.g., 60–amino acid minigenes (fig. S2B). After defining their amino acid composition, sequences were backtranslated with our Fast ALgorithm for Codon Optimization (FALCON), which rapidly generates nucleotide sequences, normalizing GC content and codon usage while removing undesired nucleotide motifs (restriction enzyme sites, homopolymer stretches, and AT- or pyrimidine-rich stretches). FALCON can process thousands of amino acid sequences within minutes, a considerable advantage over existing tools (*17*, *18*).

**Fig. 2.**
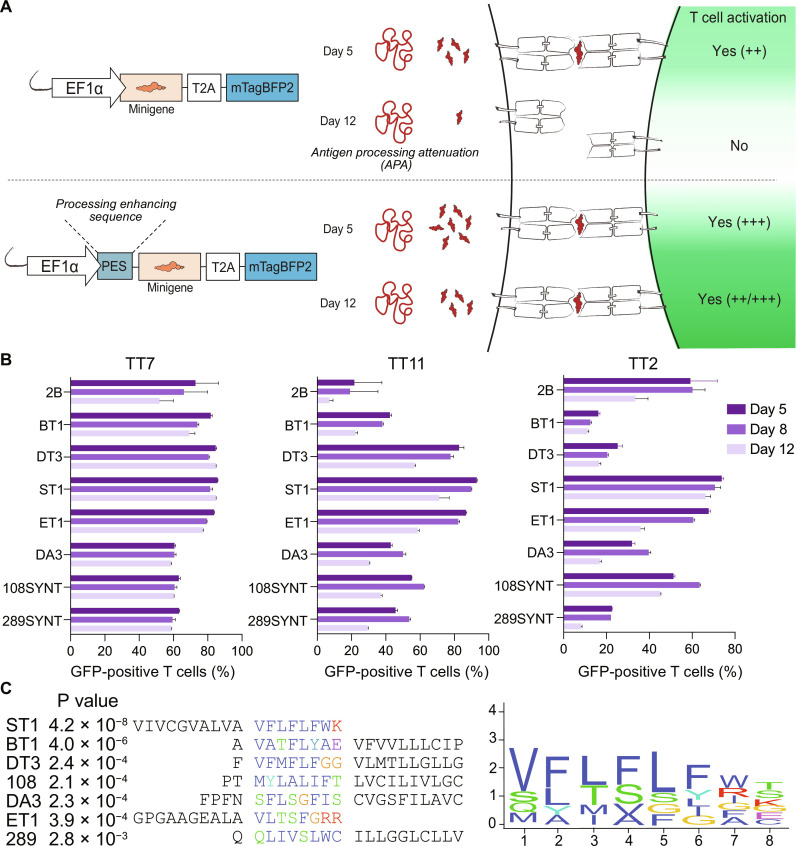
Antigen processing attenuation and processing enhancing sequences for transgenic antigen constructs. (**A**) Schematic representation illustrating the longitudinal decay of antigen processing following minigene transduction (top). However, APA is successfully overcome (bottom) by the addition of a PES. (**B**) Flow cytometric analysis of cocultures (with their respective cognate TCRs) at day 5, day 8, and day 12 following transduction with the TetX 916–1315, TetX 612–1011, and TetX 1–399 minigenes, fused to the respective PES. Error bars depict SD from 3 intra-experimental replicates; data representative of ≥2 experiments for each PES and show a single experiment in which all sequences were included. (**C**) Validated sequences from (B) were analyzed for a common motif using MEME-sorted.

Using the TT TCRs and their cognate epitope–containing minigenes from the *Clostridium tetani* tetanospasmin (TetX) protein, we observed that reporter signals accumulated over a 24-hour period without substantial cytotoxicity, with measurable readouts as early as 3 hours and maximum signal strengths attained after 16 hours of coculture. However, we encountered two issues with this approach: First, 2B-minigenes did not strongly activate the PNJE3 reporter for weaker TCRs (such as TT11; fig. S2C). Second, T cell activation decreased considerably with increasing delays between B-LCL transgenesis with the 2B construct and start of the T:APC coculture (and independently of coculture duration, which was generally fixed at 16 hours) for all class II–restricted TCRs (fig. S2C). Eliminating technical explanations such as minigene silencing and lentivirus-induced suppression of epitope presentation (fig. S2D), we concluded that antigen processing pathways are responsible for this phenomenon, which we call “antigen processing attenuation” (APA; Fig. 2A).

This severe decrease in the capacity of maintained transgenic APCs to activate cognate reporter cells creates a challenge in using culture-expanded B-LCL libraries to screen multiple TCRs for their ligands, unless all experiments could be conducted in a brief window of opportunity or sufficiently large APC libraries could be created in bulk without the need for expansion or single-cell seeding. These options being technically cumbersome, we sought to globally boost antigen presentation and circumvent APA by directing minigene-derived proteins toward specific cellular compartments. Following unsuccessful attempts to target the lysosome (fig. S2E), we observed increased, longitudinally stable T cell activation when fusing a transmembrane (TM) domain from the endoplasmic reticulum (ER)–resident B cell chaperone Bap31 (*19*, *20*) (BT1; Fig. 2B) to the minigene N terminus. We therefore screened 1152 additional TM domains of ER-resident proteins or synthetic approximations thereof (fig. S2F). From these, we identified six “processing enhancing sequences” (PES; Fig. 2B). A MEME-sorted (*21*) motif analysis of these sequences identified “VFLFLWK” from the best-performing ST1 (SYT6, TM1) sequence, which is partially conserved among the other PES (Fig. 2C).

### TCR ligand identification is rapid and robust across therapeutic indications

**Fig. 3.**
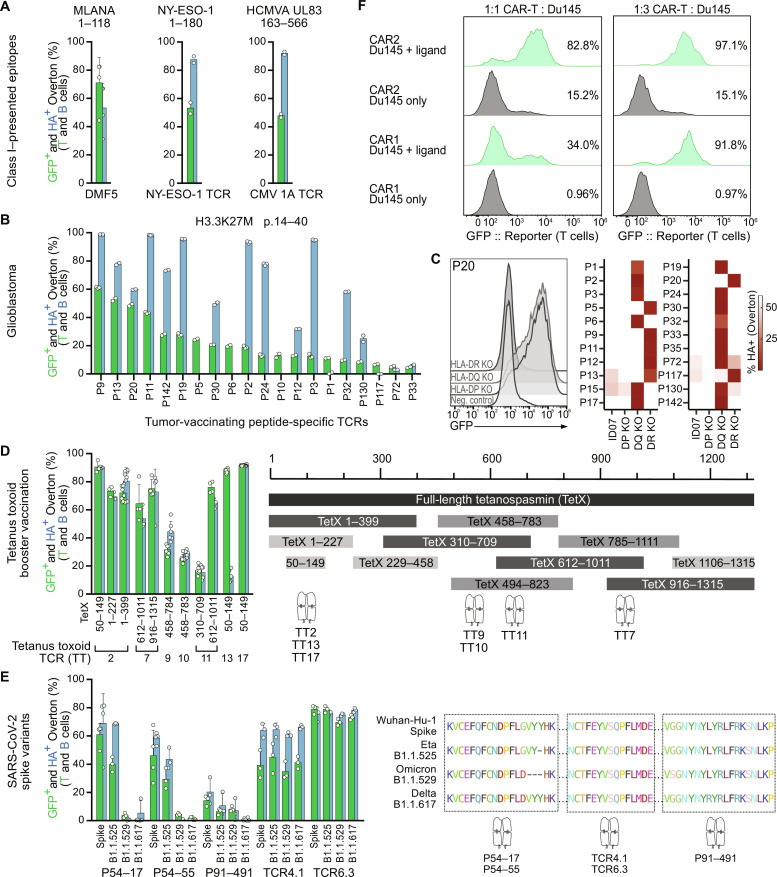
Functional assessment of T cell:ligand interactions. (**A**) PNJE3 activation by class I–restricted TCRs cocultured with HLA-matched, cognate minigene-transduced B-LCLs. (**B**) Rapid screening of putative neo-epitope reactive TCRs in H3K27M diffuse midline glioma (DMG). TCR IDs indicate their clonal frequency following ex vivo epitope-specific enrichment. Clonotypes are plotted in order of descending GFP signal following coculture with a peptide-loaded autologous B cell line. Green bars, T cell GFP expression; blue bars, B cell labeling; both signals are used to assess the significance of signal strength. (**C**) Left: Flow cytometry data for P20 TCR-transgenic PNJE3 cells in coculture with class II HLA locus-deficient autologous B-LCL loaded with cognate peptide. Right: TCR restriction analysis for the full set of deconvoluted neoepitope-specific TCRs from patient ID07. (**D**) Left: PNJE3 cells expressing TCRs of interest (TT2, TT7, TT9, TT10, TT11, TT13, and TT17) specific for class II–presented epitopes within the *C. tetani* tetanospasmin protein cocultured with DR4^+^ B-LCLs transduced with select cognate minigenes (minigene coordinates within the TetX protein listed). Right: Epitope mapping of seven tetanus toxin (TT)-reactive TCRs following coculture with the set of overlapping minigenes. (**E**) Left: Cognate validation for anti-SARS-CoV-2 TCRs. Five TCRs were cocultured with HLA-matched allogenic B-LCLs expressing Wuhan strain/variant Spike protein minigenes. Right: Sequence alignment for the four Spike protein variants depicting published epitopes of the respective TCRs. (**F**) Histograms of GFP expression by CAR1 or CAR2-transgenic PNJE3 cells following coculture with ligand expressing Du145 cells at two CAR:Du145 cell ratios (left 1:1, right 1:3). Representative data from two independent experiments.

T-FINDER’s optimization for large-scale operation should translate to less complex approaches. To confirm this, we validated several TCR sequences for reactivity to peptides or minigenes in disease-relevant settings. First, we tested TCRs specific for class I epitopes from cancer and infectious disease, including a recently identified anti-cytomegalovirus TCR (*5*). All TCRs activated the PNJE3 reporter line (Fig. 3A), confirming the platform’s sensitivity to class I presented, minigene-encoded epitopes.

We then screened clonally expanded CD4 TCRs from a patient with diffuse midline glioma (DMG) administered a neoepitope peptide vaccine (*22*) to understand whether the treatment had elicited a T cell–driven immune response; 20 of 40 tested TCR clonotypes were reactive to the histone 3 K27M vaccinating peptide (Fig. 3B). Several TCRs did not strongly activate the PNJE3 line, indicative of weaker affinity and/or avidity. Therefore, we leveraged the APC-labeling scFv to optimize our quality control and analysis pipeline, ensuring that even weak responses register significantly above background for confident detection in high-throughput assays (fig. S3A). Using single-locus HLA-deficient APC lines derived from the autologous B-LCL, we then reverse-mapped the class II alleles presenting the epitope to each TCR (fig. S3B); in this patient, peptide-reactive TCRs were restricted to the HLA-DR and HLA-DQ loci (Fig. 3C), highlighting the capabilities of our approach and breadth of the T cell response to this neo-epitope vaccine. Because of class II homozygosity, it was not necessary to further map individual presenting alleles for this patient (fig. S3C); for additional patients and more detailed clinical analyses, see the study by Boschert, Kromer, Lerner *et al.* (*23*) and (*24*).

Next, we applied T-FINDER to epitope mapping in pathogenic infections. For this, we generated a minigene pool spanning the TetX protein. By identifying which overlapping constructs activated TCR-transgenic reporter cells, we could map cognate HLA-DR4–presented epitopes within the full-length protein (Fig. 3D). Notably, we observed multiple TCRs specific for distinct epitopes within a larger minigene (TetX 612–1011), demonstrating the efficient presentation of multiple, nonoverlapping cognate targets.

Mapping immunogenic epitopes from natural infections can guide optimal first-line vaccine design. Therefore, we investigated a series of recently published (*25*, *26*) coronavirus spike (S) protein-reactive CD4 TCRs from patients with SARS-CoV-2, which had not previously been re-expressed on T cells. Lacking autologous APCs and full donor haplotype information, we used a panel of allogenic donor B-LCLs to validate five of these TCRs (Fig. 3E, left). Testing their reactivity to spike protein variant minigenes, we observed that two TCRs were unresponsive to the more recently emerged Delta and Omicron variants (Fig. 3E, right), indicating that T-FINDER can be used to promptly survey emerging variants of concern against existing T cell immunity, identify potential escape mutations, and guide the design of next-generation vaccines.

To demonstrate the flexibility and capacity of T-FINDER to assess receptors beyond canonical class I/II HLA-restricted abTCRs, we expressed a pair of chimeric antigen receptors (CAR1 and CAR2) in the PNJE3 line and cocultured these with the epithelial cell line Du145 expressing the CAR target. While both CAR reporters were strongly active at a 1:3 T:Du145 ratio, a lower abundance of APCs (1:1) enabled behavioral discrimination between the two CARs, with CAR2 having greater sensitivity to the target but measurable tonic activity in its absence (Fig. 3F).

### TCR mapping in celiac disease

**Fig. 4.**
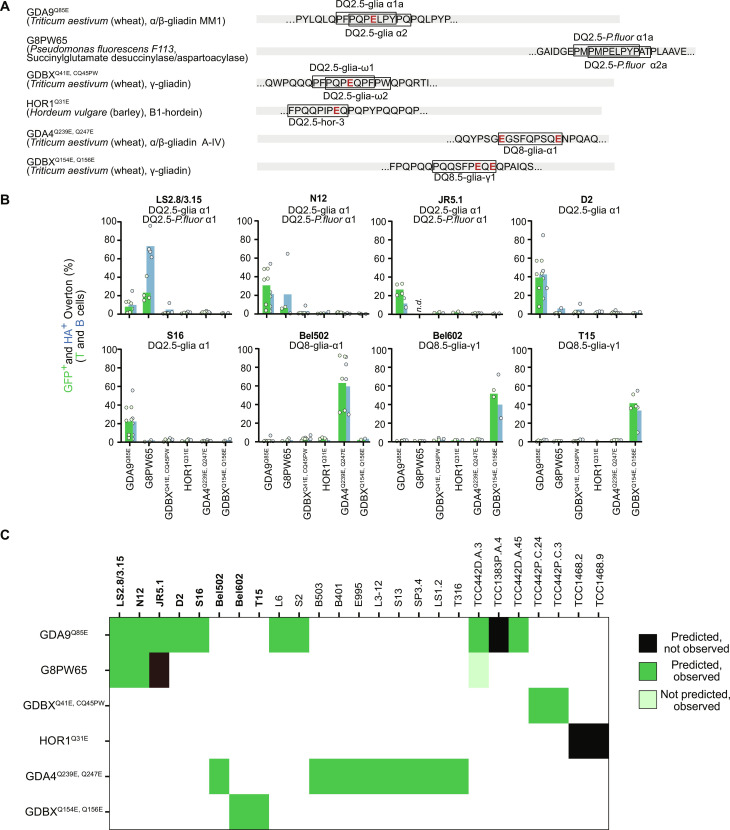
Validation of cereal gluten epitope-specific TCRs in celiac disease. (**A**) CeD peptide epitopes (black boxes) within the 400–amino acid minigenes (left labels) used in this study. Q-to-E “deamidating” mutations are indicated in red. (**B**) TCR testing using HLA-DQ matched allogenic B-LCLs. Example results of coculture between the minigenes illustrated in (A) and eight CeD TCRs with published specificities (provided above each respective plot). DQ2.5-restricted TCRs in culture with LCL0016, a DQ2.5-positive B-LCL; DQ8-restricted TCRs in culture with BOLETH cells. (**C**) Blinded screen of CeD TCRs (labeled across the top) with minigenes containing the respective CeD epitopes (labeled down the left). Black boxes indicate expected reactivities not observed in this experiment. Green boxes indicate expected (dark) and previously unreported (light) reactivities. The screen was independently performed three times and only TCRs passing QC and signal strength thresholds have been included in this summary.

Celiac disease is an immune condition resulting from T cell–driven responses to dietary gluten. The plant origin, glutamine- and proline-rich primary sequences, and post-translational deamidation of these epitopes presented potential challenges to T-FINDER’s PES-minigene system. To address this, we tested a series of published DQ2.5- and DQ8-restricted TCRs (*27*–*32*) identified in patients with celiac disease (CeD). First, we generated 400–amino acid–long minigene equivalents of known gliadin peptides (Fig. 4A), substituting glutamic acid for glutamine residues at published deamidation sites. We tested eight TCRs against this minigene panel using HLA-DQ–matched B-LCLs, observing clear activation signals for all expected interactions (Fig. 4B). We then screened a matrix of CeD TCRs against the minigene panel in an experimenter-blinded fashion. In addition to the initial set of positive controls, we matched 14 of 17 additional TCRs to their published specificities (Fig. 4C), without detecting any false positive interactions. One important aspect of CeD pathophysiology is the possibility that gluten-reactive TCRs arise from cross-reactivities with gut microbiota (*28*). Notably, we observed cross-reactivity to the g8pw65 bacterial epitope for one TCR which had not been previously documented.

### Scaling to putative target libraries for autoimmune disease

**Fig. 5.**
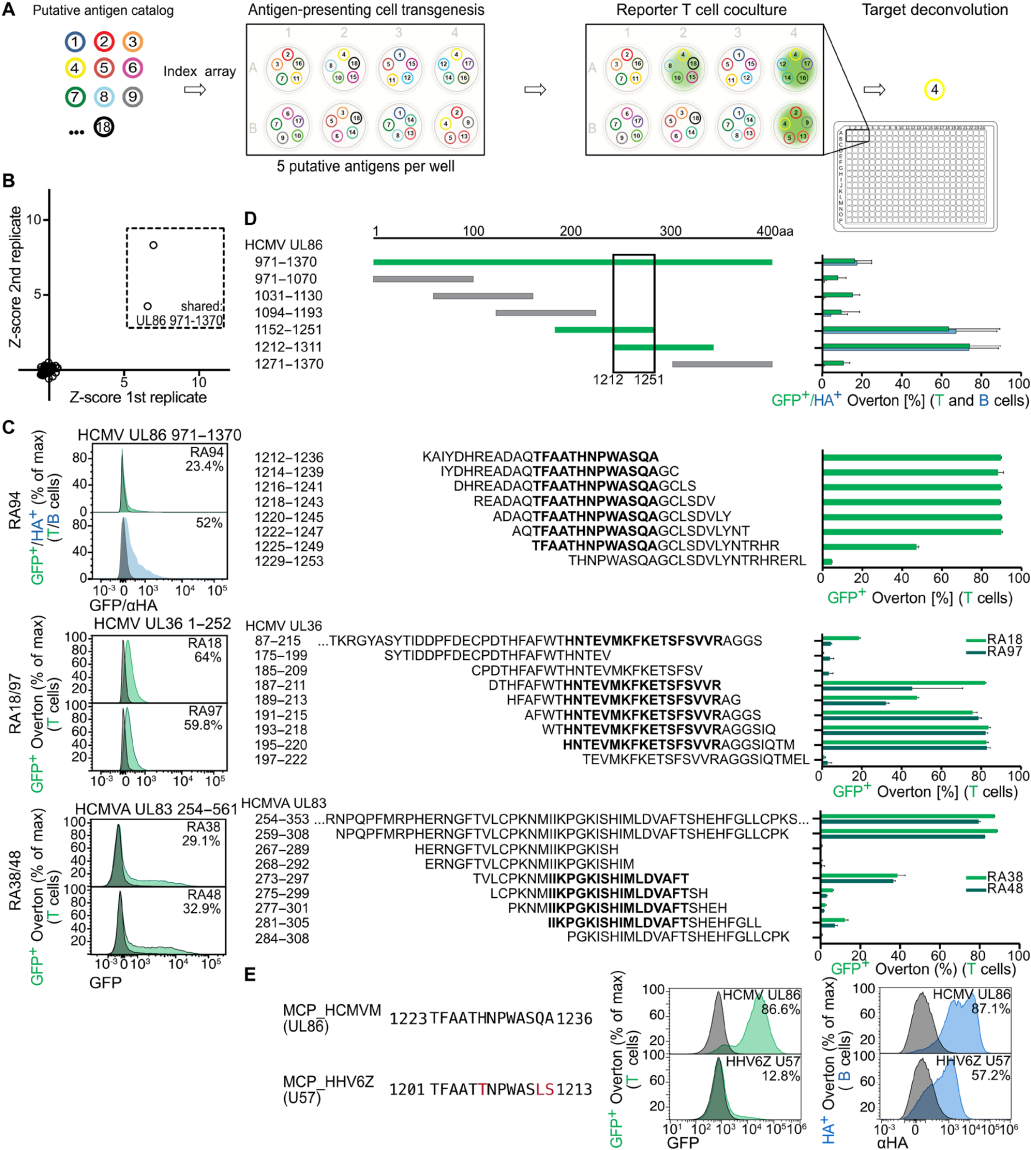
Epitope discovery, mapping, and cross-reactivity for RA TCRs. (**A**) Schematic depiction of the algorithmic antigen array principle: putative targets (numbered 1–18 in this example) are assigned to a well (8 wells in this example) such that no two co-occupy a well more than once, while each target appears multiple times in the plate. Target pools are transduced into B-LCLs and reporter T cells are added for coculture. Microscopic and/or flow cytometric analysis identifies positive wells, the combination of which immediately indicates the cognate minigene. (**B**) Identification of a fragment of the HCMV UL86 protein as a functional ligand of RA94, a RA patient–derived TCR. (**C**) Single-target validation of cognate epitope–containing minigenes for 5 RA-derived TCRs. Gray histograms, noncognate negative controls; green (GFP) and blue (αHA) histograms, coculture of the respective TCR with a minigene identified using the arrayed matrix approach. (**D**) Medium- (upper plot) and high- (lower three plots) resolution minigene scanning for epitope-containing regions within cognate 400–amino acid minigenes. Bold sequences indicate the minimal epitopes of the respective TCRs in the adjacent plots from (C). Plots shown are representative examples of ≥2 independent experiments for each TCR. (**E**) Cross-reactivity between the RA94 mapped minimal epitope, provided as an in vitro loaded peptide (top), and homologous peptide from a genomically integrated herpesvirus (bottom). Representative histograms from one of four independently performed experiments.

We next expanded the scale of T-FINDER to many putative targets using a minigene library containing several rheumatoid arthritis (RA)–associated autoantigens, as well as 162 viral and bacterial minigenes associated with common infections and/or autoimmunity, intended as a counterscreening tool for autoreactive TCRs (and including TetX minigenes as positive controls, see table S1 for details). Reporter T cells can be activated by cognate APCs even when the latter are diluted by nontargets (fig. S4A); therefore, we designed an arrayed matrix which would allow us to definitively identify the correct target without downstream sequencing. For this, minigenes are distributed among wells according to a set-generating algorithm which assigns replicates such that no two putative targets co-occur in more than one well of the matrix. In this way, the wells containing activated T cells create a unique combination that correctly identifies the target minigene (Fig. 5A).

Screening our TT TCRs as positive controls and 11 RA patient–derived TCRs (*33*) (with targets unknown to the experimenters) against the array in duplicate, we confirmed all control TCR targets (fig. S4B) and identified multiple positive wells sharing a single target for three RA TCRs (RA94, Fig. 5B, and RA18/RA97; fig. S4C). We could validate all deconvoluted RA targets in single-minigene coculture (Fig. 5C and fig. S4D), with RA18 and RA97 both reacting to the UL36 tegument protein of herpes simplex virus1 and RA94 reacting to the UL86 major capsid protein of the human cytomegalovirus. These TCRs were each identified in one of three donors, which may reflect persistent antiviral responses and the possible recruitment of activated T cells to pro-inflammatory tissue sites in autoimmune patients.

### Minimal TCR epitopes can be rapidly mapped via successively smaller antigenic fragments

Following cognate minigene identification, one logical next step is to identify that TCR’s minimal epitope within it. As a relatively fast, inexpensive, and flexible alternative to overlapping peptide libraries, we used progressively smaller minigenes. In this way, we mapped minimal epitopes of the previously deconvoluted RA-derived TCRs, as well as those of two additional RA-derived TCRs (RA38 and RA48) whose targets did not initially pass quality control thresholds in the arrayed screen but could be validated in single-minigene coculture (Fig. 5D).

Because TCR specificities cover an “epitope space” with a range of affinities (*34*, *35*), their cross-reactivity to multiple peptides is both possible and expected. Amino acid epitope resolution allows us to revisit the concept of epitope mimicry in autoimmune disease, in this case by enabling a more precise homology search between viral targets and potentially cross-reactive human proteins. As an example, a BLAST homology search of the minimal epitope for RA94 against the human reference proteome identified a similar peptide encoded by a genomically integrated viral capsid protein (*36*). This fragment also activates the RA94 TCR (Fig. 5E). Continuing work mapping such epitope spaces will support the connection between the presence of apparently antiviral TCRs in autoimmune tissues and their contribution to inflammation via additional, self-derived targets.

## DISCUSSION

T-FINDER is a flexible TCR deconvolution platform that features a highly sensitive promoter capable of transducing signals from TCRs, CARs, and related receptors (e.g., B cell receptors, γδ TCRs, and natural killer cell receptors). The importance of sensitivity has been highlighted by several studies establishing similar systems (*37*–*39*). In particular, the J76-based TCR triple-reporter cell line (*38*) is interesting because of its ability to mechanistically dissect individual TCR signaling pathways contributing to T cell activation. Our approach was instead to integrate all activation-related activity into a single, compact locus control region, with the goal of optimizing the total signal-to-noise ratio between an inactive and a cognate epitope–activated T cell from a single reporter cassette. To this end, we designed custom promoters which accumulate transcriptional activity with each additional engaged signaling pathway, and which uniquely include multiple binding sites for the lymphocyte-specific transcription-activating Bob1/Oct-1/2 complex (*40*). We observe little to no aberrant signal in the absence of TCR-cognate epitopes, indicating that, while highly sensitive, our promoter is not susceptible to false-positive or tonic TCR signals. By engineering the reporter line, rather than the presenting cell, T-FINDER remains compatible with a variety of antigen presentation options including multiple cell types, HLA classes, and non-HLA ligands, the latter supported by our observations with CAR constructs (Fig. 3F). When using autologous B-LCL as APCS, T-FINDER interrogates an individual’s entire HLA repertoire—a unique capability among deconvolution approaches.

Computational approaches which can predict cognate TCR sequences for a known epitope exist (*41*); however, the discovery of previously unidentified ligands for a given TCR sequence presents a much greater in silico challenge, and the accuracy of these models is limited by the trustworthiness of their training sets: many publicly available ligandomes are either based on TCRβ chain sequencing only (rather than complete αβ pairs) and/or have not been functionally validated. Early attempts to use more than 15 class I TCR:autoantigen pairs obtained from public databases as positive controls for our system failed to activate Jurkat cells as minigenes, while only one provided a functional response with high concentrations of in vitro loaded peptides (sample data shown in fig. S5). In contrast, T-FINDER inherently deconvolutes and functionally validates TCR:ligand pairs and thus can be used to increase the size and quality of training data sets for predictive models.

Measuring the epitope-specific activation of primary T cells is challenging because of their reduced viability ex vivo and the presence of tolerized or exhausted cells, which may be lost during in vitro expansion processes. Certain situations will still require an enrichment step for ligand (set)–reactive TCRs, such as when donor samples are only available from the peripheral blood, which particularly for CD4 T cells has very complex TCR repertoires (*42*). However, re-expressing relevant TCRs (based on clonal expansion or anatomical location) provides a clear advantage with respect to the yield of functional cognate pairs.

The high-throughput capacity of T-FINDER is made possible using 400–amino acid–long minigene constructs. While the feasibility of genetically encoded TCR epitopes has been previously demonstrated (*4*, *43*) their applications have been largely limited to class I HLA presentation and shorter (<100 amino acid) protein fragments. One explanation for this is that longer minigenes yield lower TCR-cognate peptide dosage on the APC surface, which we are now able to detect with the increased sensitivity of the PNJE3 reporter line. In addition, our observations here suggest that longer minigenes, although preferable because of their ability to encode multiple, HLA-agnostic and naturally processed epitopes, underperform because of inefficient class II processing. We demonstrate on a larger scale that this is attributable to APC-intrinsic antigen processing control (Fig. 2A) and have overcome this limitation by identifying and fusing an N-terminal PES, which results in robust presentation of multiple epitopes encoded within 400–amino acid constructs (e.g., the targets of TT9, TT10, and TT11 in Fig. 3D). To this effect, using genetically encoded TCR ligands rather than loaded peptides leverages the cellular antigen-processing machinery, increasing physiological relevance by offering the APC protein moieties to be naturally cleaved and loaded onto HLA. This approach allows APCs to dictate peptide length and composition, and can identify epitopes without e.g., mass spectrometric ligandome studies (*44*). In addition to endogenous antigen processing, T-FINDER can accommodate posttranscriptional modifications with minor adaptations (e.g., genetically introducing deamidated residues or expressing modifying enzymes in the B-LCL). Hence, T-FINDER’s antigen libraries simultaneously provide greater screening breadth and depth relative to other approaches.

With several alternatives to TCR and epitope discovery currently available, but none satisfying our strict requirements for potency and flexibility, we have established a multicomponent system that can be adapted to serve many needs in T cell–based applications. T-FINDER uncovers biological insights at a pace that was previously not possible, enabling and improving the development of immune-specific diagnostics and therapeutics.

## MATERIALS AND METHODS

### Cell lines

T cell reporter lines with and without TCRs were cultured in GlutaMAX supplemented RPMI 1640 medium further modified with 10% heat-inactivated fetal bovine serum (FBS), penicillin (100 U ml^−1^), streptomycin (100 μg ml^−1^), 1 mM sodium pyruvate, and 55 μM β-mercaptoethanol. TCR signaling reporter lines were additionally cultured with blasticidin (10 μg ml^−1^), and TCR-transgenic lines were selected using puromycin (1 μg ml^−1^). The human lymphoblastoid line BOLETH was obtained from Sigma-Aldrich (#88052031-1VL). B-LCLs were cultured in GlutaMAX supplemented RPMI 1640 medium with 10% FBS, penicillin (100 U ml^−1^), streptomycin (100 μg ml^−1^), 1 mM sodium pyruvate, 1x MEM Non-Essential Amino Acids Solution, and 55 μM β-mercaptoethanol. HEK293T cells were cultured in high-glucose GlutaMAX supplemented Dulbecco’s modified Eagle’s medium (DMEM) containing 10% heat-inactivated FBS, penicillin (100 U ml^−1^), and streptomycin (100 μg ml^−1^). DPBS without calcium or magnesium and 0.05% Trypsin-EDTA was used for passaging. All human cell lines were cultured at 37°C with 5% CO_2_ in a humidified atmosphere.

### Primary cell culture and B-LCL immortalization

For Epstein-Barr virus–induced immortalization of donor-derived B cells, PBMCs were isolated from buffy coats using 50-ml Leucosep centrifuge tubes, Ficoll Paque Plus, and ACK erythrocyte lysis buffer. B cells were extracted by negative selection with the EasySep Human B Cell Isolation Kit (StemCell Technologies, #17954). EBV-containing supernatants from B95–8 cells were added to purified B cells supported by holo-transferrin (30 μg ml^−1^) and CpG (2.5 μg ml^−1^; ODN2006). Established lines were haplotyped by DKMS Life Science. The primary tumor cell line used for antigen presentation to CAR1 and CAR2 was kindly provided by Janssen.

### Development of the reporter construct and PNJE3 reporter line

Synthetic TCR signaling-response promoter sequences (table S1) were cloned 5′- of the reporter cassette in a Sleeping Beauty transposon backbone. This cassette, protected by core insulator sequences, contains an HA-tagged αCD19 [B43 (*45*)] scFv sequence followed by a furin cleavage site, T2A viral peptide, and superfolder GFP (*9*). The 3′-UTR of human IgG1 was used to increase transcript stability and provide a poly-adenylation signal. Following this inducible cassette and a core insulator sequence, a constitutively active PGK promoter drives expression of CD28 and a blasticidin resistance gene. A separate plasmid containing the transposase was cotransfected (1:5). All DNA sequences were synthesized by Twist Bioscience. To generate the T cell reporter line, 2.5 × 10^6^ TCR-deficient Jurkat or SKW-3 cells were electroporated with plasmid DNA using the Neon Transfection system (Thermo Fisher Scientific). Single-cell clones were expanded under selection (blasticidin (8 or 10 μg ml^−1^ ) for 2 weeks before measuring TCR-inducible reporter activity.

### Design and construction of TCR reporter cells

TCRs were generated by custom synthesis (Twist Bioscience) in a third-generation pLVX-EF1α-IRES-Puro lentiviral vector (Takara/Clontech). Following the TransIT-Virusgen Transfection (Mirus, #MIR6700) protocol, HEK293T cells were cotransfected with the pMD2.G (VSV-G envelope) and psPAX2 (packaging) plasmids. Jurkat and SKW-3 reporter cells were transduced with filtered viral supernatant and polybrene (1 μg ml^−1^) 48 hours after transfection. Viral supernatant was replaced with fresh medium after 24 hours and selected with puromycin (1 μg ml^−1^) 96 hours after transduction. CAR1 and CAR2 were provided as filtered viral supernatants by Janssen and processed as described above.

### TCR sequences

The patient with glioma (ID07) received H3K27M-vac after providing written, signed informed consent at the University Hospital of Mannheim with treatment and sample use approved by the institutional review board. Other sequences were reconstituted as full-length TCRs from published gene segment usage and CDR3 information or identified following TCR sequencing of pseudonymized cellular material upon provision of informed consent for experimental purposes.

### Design, cloning, and transgenesis of genetically templated antigens

Epitope-containing minigene sequences were generated by custom synthesis (Twist Bioscience). Smaller fragments were amplified by PCR from minigenes with custom primers (Integrated DNA Technologies) using Q5 high-fidelity DNA Polymerase. Each construct was flanked by unique SpeI and BamHI sites to enable efficient cloning into a pLVX-EF1α-IRES-Puro lentiviral vector, which places the respective fragment upstream of an in-frame, T2A-linked mTagBFP2 (*46*) sequence. Lentiviral transduction included an additional pre-incubation step of the B-LCLs with 4 nM BX-795 hydrochloride. Viral supernatant was replaced with fresh medium 24 hours after transduction. Seventy-two hours after this medium change, the expression was verified by flow cytometric analysis of BFP and functional assays were performed.

### Codon-optimized backtranslation

An archived release of the FALCON script is available at https://doi.org/10.5281/zenodo.10037761.

### In vitro loaded peptides

Peptides were generated by custom peptide synthesis (Biomatik) or provided as peptide pools (JPT or Miltenyi BioTec, see SI for details). Peptides were resuspended at a stock concentration of 10 mg ml^−1^ in 10% DMSO and stored at −80°C. Peptide concentrations were adjusted in a dilution series with DMSO and an identical volume was added to B-LCL cells. The final concentrations of peptides used in cocultures are shown in Fig. 1 and fig. S1.

### Peptide and protein electroporation

Full-length inactivated tetanus toxoid (Uniprot ID P04958, 1315 aa) was kindly provided by Janssen, while cognate peptides were generated by custom synthesis (Biomatik). Both were resuspended at a stock concentration of 10 mg ml^−1^ in 10% DMSO and stored at −80°C. Per electroporation, 5 × 10^5^ B-LCL were washed with DPBS (without Ca^2+^ and Mg^2+^) and resuspended at 5 × 10^7^ cells ml^−1^ in 0.9% DPBS, adding full-length protein or peptide to the required final concentration. After electroporation (1300 V, 30 W, 1 P), cells were resuspended in complete, supplemented medium at 2 × 10^6^ cells ml^−1^. Electroporated cells recovered for 1 hour at 37°C, 5% humidified CO_2_ before performing functional assays.

### T cell reporter stimulation and coculture assays

Ligand-independent reporter cell activation was assessed by overnight stimulation with 6.25 μg ml^−1^ Ultra-LEAF Purified anti-human CD3 (OKT-3, BioLegend, #317326). Cells were incubated in antibody-coated wells and TCR responses were measured by flow cytometric analysis of GFP expression after 16 hours. For coculture experiments, 10^5^ reporter cells were seeded in U-bottom 96-well plates with fresh complete medium either 4 days after TCR transduction or following 2 additional weeks of selection [puromycin (1 μg ml^−1^)]. After 1-hour recovery in fully supplemented medium, APCs were added. Minigene-expressing APCs were cocultured with reporter cells 4 days after transduction. In both approaches, APCs were combined with reporter cells at a 1:1 or 2:1 ratio in a final volume of 200 μl and incubated overnight (16 hours) at 37°C, 5% CO_2_ in a humidified atmosphere. Flow cytometry was used to assess TCR responses to HLA-presented epitopes through GFP expression and the B cell-bound HA-tagged αCD19 scFv fragment. T and B cells were identified using surface staining for CD2 and CD20, respectively. BFP was used to evaluate minigene translation efficiency.

### Flow cytometry analysis

The following antibodies from BioLegend were diluted in flow cytometry buffer (DPBS, 2% FBS, 2 mM EDTA): PerCP-Cy5.5-conjugated anti-human CD2 (RPA-2.10, 0.25 μg ml^−1^, #300216), PE-Cy7-conjugated anti-human CD3e (UCHT1, 0.75 μg ml^−1^, #300420), Pacific Blue or PE-Cy7-conjugated anti-human CD20 (2H7, 1 μg ml^−1^, #302320 or #302312), APC-Cy7 or PE-Cy7-conjugated anti-human CD69 (FN50, 0.5 μg ml^−1^, #310914 or #310912), and APC anti-HA.11 epitope tag (16B12, 0.5 μg ml^−1^, #901524). BV395-conjugated CD20 (2H7, 0.25 μg ml^−1^, #563782) was purchased from BDbiosciences. Propidium Iodide Ready Flow Reagent (Thermo Fisher, #R37169) was used as viability dye. GFP and BFP were additionally analyzed to identify TCR activation and minigene translation efficiency. Cells were washed before staining for 20 min at 4°C in the dark. Samples were then washed and analyzed using a BD FACSAria Fusion or BD FACSAria II cell sorter, or in high throughput using a Sony ID7000 spectral analyzer. Data analysis was performed using FlowJo (10.6.1). For single-variable analysis, the percentage of positive cells was calculated as the integrated area under the histogram not overlapping with control samples using FlowJo’s population comparison (Overton) method.

### Design, coculture, and imaging of multiplexed arrays

Following each algorithmically-generated pipetting scheme (archived v1 release available on Zenodo https://doi.org/10.5281/zenodo.10040021), 5 independent minigenes were combined in each well of a low-DNA binding 96-well plate. 65 ng of each minigene resulted in a total of 0.325 μg of lentiviral vector in each 96-well. As described above, TransIT-Virusgen Transfection reagent was used, including 33 ng pMD2.G and 50 ng psPAX2 per well. Forty-eight hours after transfection of virus-producing 293 T cells, 5 × 10^4^ B-LCL cells were treated with BX-795 hydrochloride and polybrene (1 μg ml^−1^) for 15 min, before adding viral supernatant. For microscopy, B-LCL medium was exchanged for FluorBrite DMEM (Thermo Fisher, #A1896701) supplemented with 5% FBS, penicillin (100 U ml^−1^) and 100 μg ml^−1^, 1 mM sodium pyruvate, 1X MEM Non-Essential Amino Acids, and 55 μM β-mercaptoethanol 4 days after transduction. Transduced B-LCL cells of each 96 well were split into two wells of a clear-bottom 384-well plate. In an equal ratio, 2.5 × 10^4^ reporter cells selected for a TCR of interest were added to each 384 well. GFP expression was imaged following 7 hours of coculture. Using the automatic scan function of the Nikon Eclipse Ti2 microscope, GFP images were recorded individually for each 384 well at 10x magnification (500 ms). After removing the background, a macro for the software Fiji-ImageJ was applied to extract the GFP-positive area of each individually measured well. *Z*-scores were calculated as the number of standard deviations of each well above or below the mean using the distribution of all wells in a given experiment; geometric means from technical replicates were calculated to determine the signal threshold for positive wells. Minigenes shared among positive wells were identified as cognitive minigenes for the investigated TCR(s).
