# Supplementary material for: T-FINDER: A highly sensitive, pan-HLA platform for functional T cell receptor and ligand discovery
Source: Sci Adv. 2024 Feb 2;10(5):eadk3060. doi: 10.1126/sciadv.adk3060 (PMC10836725; doi:10.1126/sciadv.adk3060)
Supplement: Supplementary file 1 — Supplementary Text Figs. S1 to S5 [file sciadv.adk3060_sm.pdf]

Science Advances
AAAS

Supplementary Materials for

T-FINDER: A highly sensitive, pan-HLA platform for functional T cell receptor and ligand discovery

Miray Cetin *et al.*

Corresponding author: John M. Lindner, lindner@bio.mx

Sci. Adv. **10**, eadk3060 (2024)
DOI: 10.1126/sciadv.adk3060

The PDF file includes:

Figs. S1 to S5

Other Supplementary Material for this manuscript includes the following:

Table S1

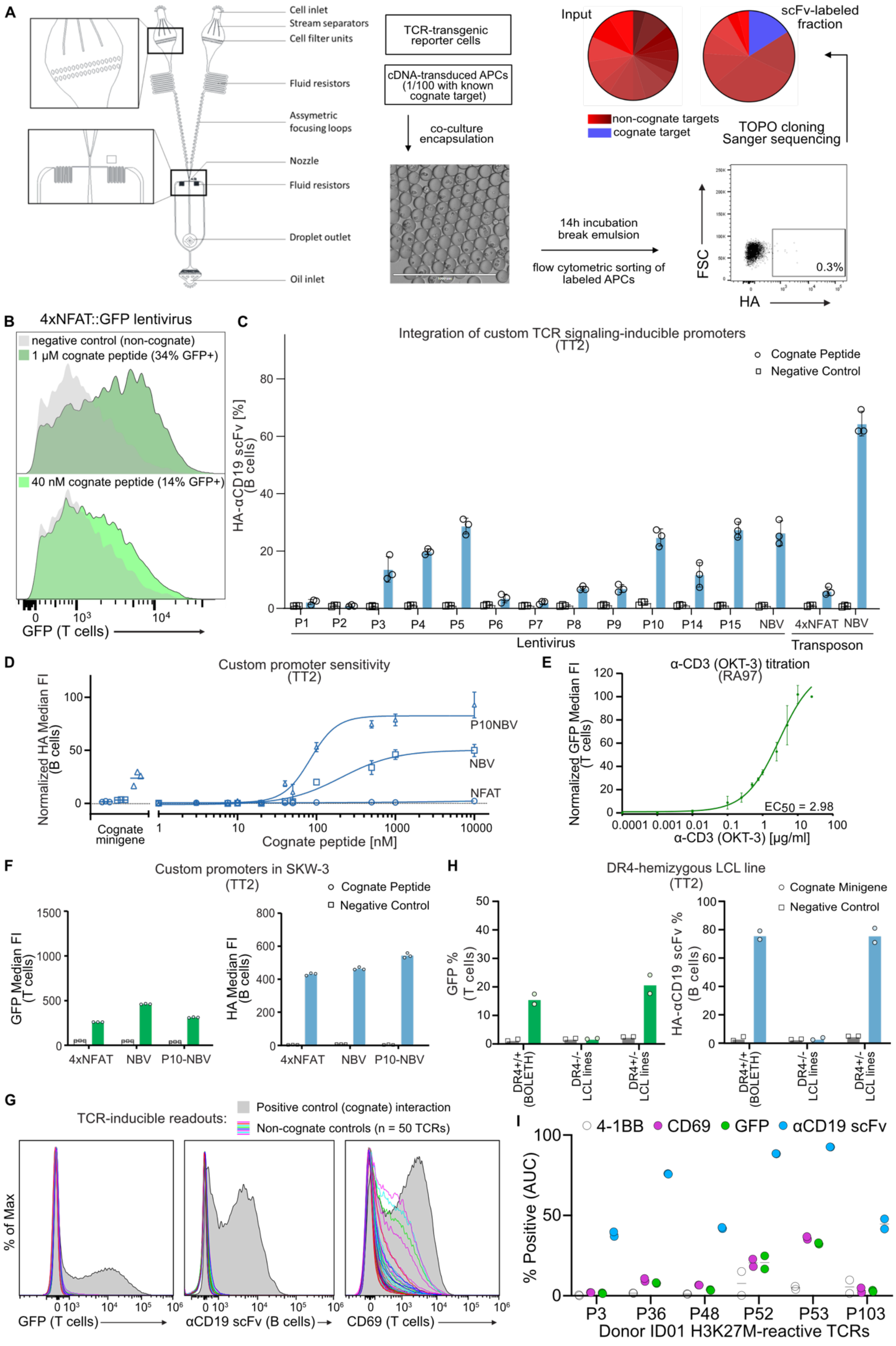

A
Cell inlet
Stream separators
Cell filter units
Fluid resistors
Assymetric focusing loops
Nozzle
Fluid resistors
Droplet outlet
Oil inlet
TCR-transgenic reporter cells
cDNA-transduced APCs (1/100 with known cognate target)
co-culture encapsulation
14h incubation break emulsion
flow cytometric sorting of labeled APCs
Input
scFv-labeled fraction
non-cognate targets
cognate target
TOPO cloning Sanger sequencing
FSC
HA
0.3%
B
4xNFAT::GFP lentivirus
negative control (non-cognate)
1 µM cognate peptide (34% GFP+)
40 nM cognate peptide (14% GFP+)
GFP (T cells)
C
Integration of custom TCR signaling-inducible promoters (TT2)
Cognate Peptide
Negative Control
HA-αCD19 scFv [%] (B cells)
P1 P2 P3 P4 P5 P6 P7 P8 P9 P10 P14 P15 NBV 4xNFAT NBV
Lentivirus
Transposon
D
Custom promoter sensitivity (TT2)
Normalized HA Median FI (B cells)
P10NBV
NBV
NFAT
Cognate minigene
Cognate peptide [nM]
E
α-CD3 (OKT-3) titration (RA97)
Normalized GFP Median FI (T cells)
EC50 = 2.98
α-CD3 (OKT-3) [µg/ml]
F
Custom promoters in SKW-3 (TT2)
Cognate Peptide
Negative Control
GFP Median FI (T cells)
HA Median FI (B cells)
4xNFAT NBV P10-NBV
H
DR4-hemizygous LCL line (TT2)
Cognate Minigene
Negative Control
GFP % (T cells)
HA-αCD19 scFv % (B cells)
DR4+/+ (BOLETH)
DR4-/- LCL lines
DR4+/- LCL lines
G
TCR-inducible readouts:
Positive control (cognate) interaction
Non-cognate controls (n = 50 TCRs)
% of Max
GFP (T cells)
αCD19 scFv (B cells)
CD69 (T cells)
I
4-1BB CD69 GFP αCD19 scFv
% Positive (AUC)
P3 P36 P48 P52 P53 P103
Donor ID01 H3K27M-reactive TCRs

Fig. S1. TCR activation reporters and minigene antigen constructs. (**A**) Design and demonstration of a high-throughput, practical application for APC labeling via activated T cell scFv secretion. The schematic on the left illustrates design principles for a microfluidic device used to co-encapsulate individual reporter T cells with transgenic APCs. The experimental pipeline on the right provides sample data demonstrating the enrichment of a positive control cognate target to detectable levels when present at 1% frequency in input. (**B**) Signal-to-noise ratios of a 4xNFAT promoter as an integrated lentiviral cassette in Jurkat cells in response to T-cell activation with cognate peptide loaded *in vitro* at high (top) and low (bottom) concentrations. (**C**) Labeling of cognate B-LCLs (via αCD19 scFv secreted by activated reporter T cells) for a series of bespoke TCR signaling-inducible promoters. Promoters with strongest activity were stably integrated as transposons (rightmost bars), together with a 4xNFAT binding sequence promoter for comparison. (**D**) Minigene and peptide dilution curves (HA-tagged αCD19 scFv) of a cognate epitope for three transposon-integrated inducible reporter cassettes (bulk T cell population prior to single-clone selection). (**E**) Titration curve for TCR-independent reporter T cell activation of one rheumatoid arthritis-derived TCR using an αCD3 antibody (OKT-3). (**F**) Inducible promoter activity (lentiviral integration) in SKW-3 cells. (**G**) T-FINDER readouts (left, T cell GFP expression; middle, B-cell αCD19 labeling) of reporter T cell activation (cognate minigene co-culture, solid histograms) and background noise (negative control TCRs with known targets not expressed by the APCs, colored lines), relative to T cell CD69 surface expression as a readout (right). (**H**) Bar graphs showing TT2-transgenic reporter T cell activation (left, GFP expression; right, B-cell labeling) following co-culture with a homozygous HLA-DR matched cell line (BOLETH, DR4+/+), a DR4-negative B-LCL, and a hemizygous (DR4+/-) HLA-haplotyped LCL line pulsed with cognate peptide. (**I**) Activation levels of PNJE3 reporter cells (TCRs with cognate peptide-loaded autologous B-LCL) with activation-induced markers 4-1BB (CD137), CD69, and T-FINDER readouts GFP and HA-tagged αCD19 scFv; AUC, area under the curve (Overton).

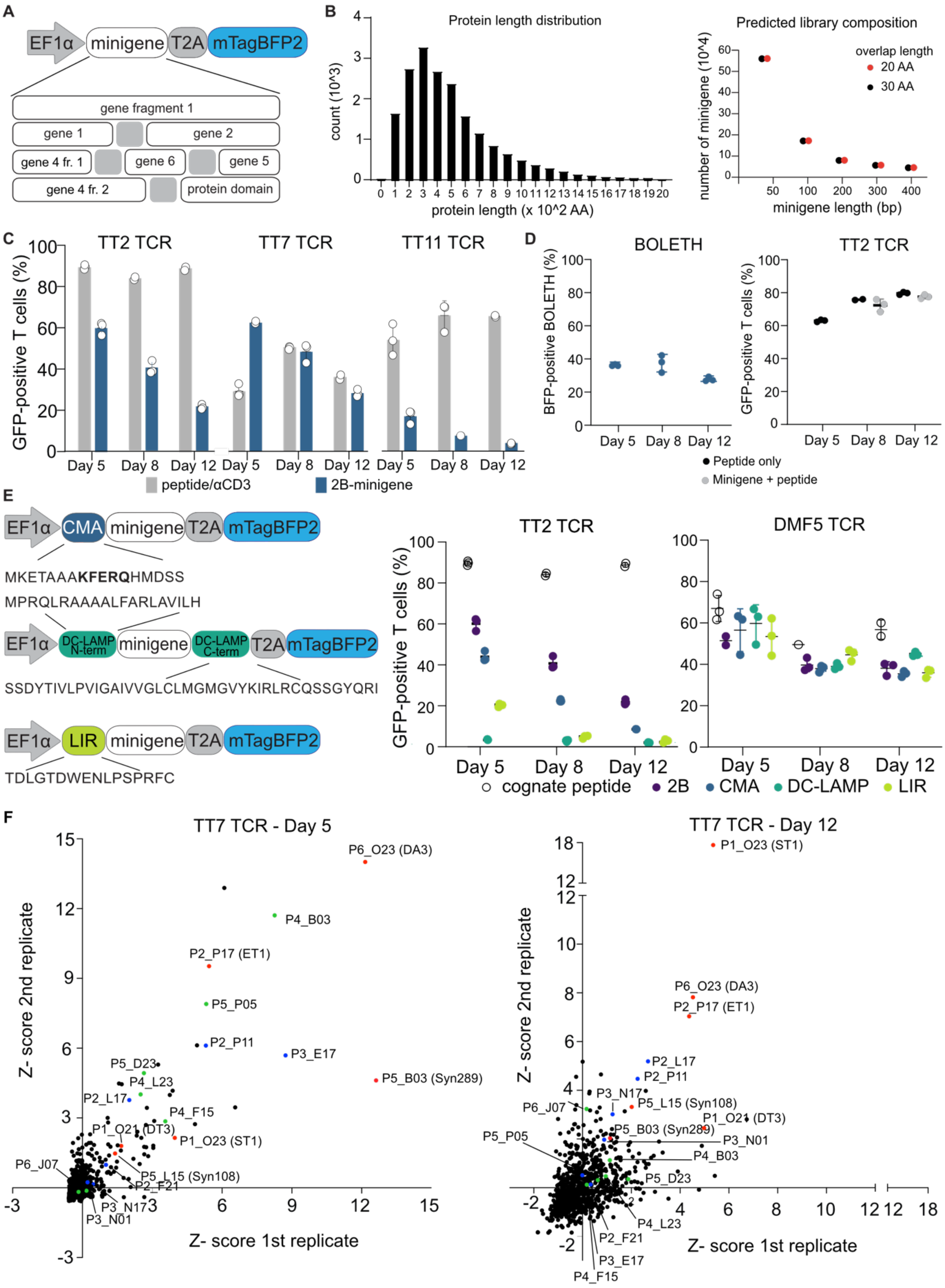
A
EF1α
minigene
T2A
mTagBFP2
gene fragment 1
gene 1
gene 2
gene 4 fr. 1
gene 6
gene 5
gene 4 fr. 2
protein domain
B
Protein length distribution
count (10^3)
protein length (x 10^2 AA)
Predicted library composition
number of minigene (10^4)
minigene length (bp)
overlap length
20 AA
30 AA
C
TT2 TCR
TT7 TCR
TT11 TCR
GFP-positive T cells (%)
Day 5
Day 8
Day 12
peptide/αCD3
2B-minigene
D
BOLETH
BFP-positive BOLETH (%)
TT2 TCR
GFP-positive T cells (%)
Peptide only
Minigene + peptide
E
CMA
MKETAAAKFERQHMDSS
MPRQLRAAAALFARLAVILH
DC-LAMP N-term
DC-LAMP C-term
SSDYTIVLPVIGAIVVGLCLMGMGVYKIRLRCQSSGYQRI
LIR
TDLGTDWENLPSPRFC
TT2 TCR
DMF5 TCR
GFP-positive T cells (%)
cognate peptide
2B
CMA
DC-LAMP
LIR
F
TT7 TCR - Day 5
Z- score 2nd replicate
Z- score 1st replicate
P6_O23 (DA3)
P4_B03
P2_P17 (ET1)
P5_P05
P2_P11
P3_E17
P5_B03 (Syn289)
P5_D23
P4_L23
P2_L17
P4_F15
P1_O21 (DT3)
P1_O23 (ST1)
P6_J07
P5_L15 (Syn108)
P2_F21
P3_N17
P3_N01
TT7 TCR - Day 12
P1_O23 (ST1)
P6_O23 (DA3)
P2_P17 (ET1)
P2_L17
P2_P11
P3_N17
P5_L15 (Syn108)
P6_J07
P1_O21 (DT3)
P5_B03 (Syn289)
P5_P05
P3_N01
P4_B03
P5_D23
P4_L23
P2_F21
P3_E17
P4_F15

Fig. S2. Minigene structure and antigen expression constructs with their strategies. (**A**) Schematic representation of the lentiviral vector for expression of an antigen-presenting library, driven by an EF1α promoter. The ORF contains a minigene linked to mTagBFP2 with a T2A peptide. Minigene sequences can be composed of a gene fragment, multiple short genes linked together, genes linked with gene fragments, or a full-length gene linked to a "space-filling" protein domain. (**B**) Analysis of protein lengths across the human proteome and predicted number of elements in a proteome-wide minigene library considering minigene length and tiling density (20 aa overlap in black and 30 aa overlap in red). (**C**) Longitudinal co-culture experiment for class II HLA-presented epitopes using the TetX 1 – 399 minigene and TT2 TCR, or TetX 612 – 1011 with the TT11 and TT7 TCRs. Positive controls (gray bars) were 1 μM Tet93 peptide (TT2), 1 μM Tet614 peptide (TT11), or 2.5 μg/ml α-CD3 (TT7). (**D**) mTagBFP2 expression of the transduced BOLETH cells used at each time point. (I) Class II HLA antigen loading control experiment; grey circles, T-cell activation using minigene-transgenic BOLETH cells supplemented with pulsed peptides; black circles, co-culture with freshly peptide-pulsed B-LCLs. (**E**) Schematic of class II processing constructs (left) used in this study. Flow cytometric analysis of co-cultures (right) at day 5, day 8, and day 12 after transduction with CMA, DC-LAMP, and LIR constructs for minigene expression. TCR:minigene pairs used: TT2:TetX 1 - 399, DMF5 – MLANA^{A27L}. 1 μM Tet93 and 10 μM MART-1, respectively, were used as positive controls for reporter cell activation, expressed as the percentage of GFP positive T cells following co-culture. (**F**) Results of a plate-based screen of ER_TM domain library elements using the TetX 916 – 1315 minigene and cognate TT7 TCR. Left panel, results obtained at day 5; right panel, results at day 12 post-transduction. The intensity of the GFP signal is represented as the Z-scores (over all tested constructs) of two replicates (x- and y-axes). Red dots indicate colonies selected for validation/follow-up.

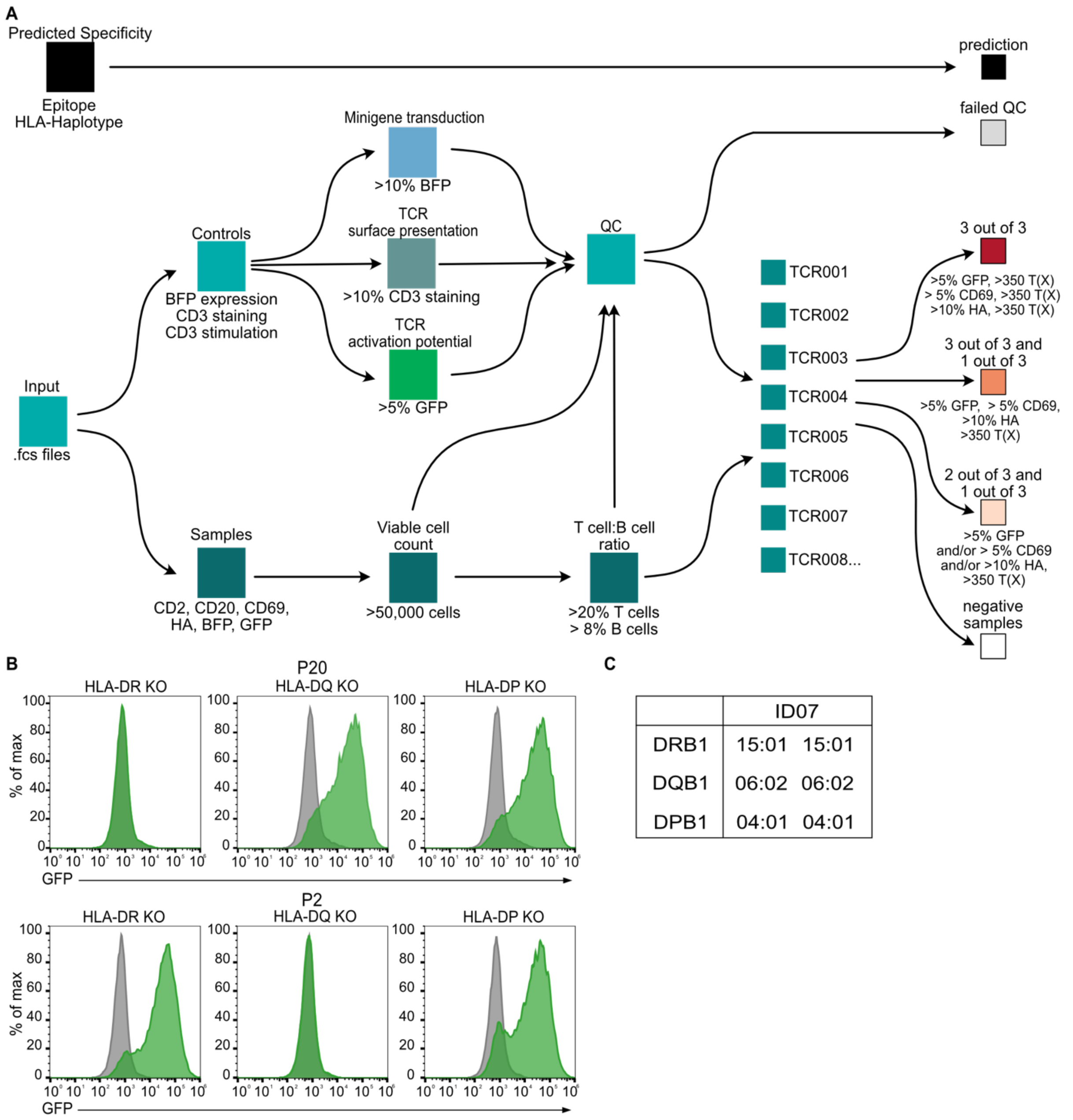

	ID07	
DRB1	15:01	15:01
DQB1	06:02	06:02
DPB1	04:01	04:01

Fig. S3. TCR Analysis pipeline and HLA restriction mapping of glioma peptide-reactive TCRs. (**A**) Schematic of the parameters analyzed to ensure robust and high-quality validation calls on large, blinded TCR:target screens. The following experimental parameters are considered: successful TCR expression by reporter cells (>10% of CD3-positive T cells), activation potential upon ligand-independent αCD3 stimulation (>5% of GFP-positive T cells), successful minigene expression by B-LCLs (>10% of BFP-positive B cells), and acceptable T:B ratios in the co-culture. Only samples that pass all determined QC thresholds are eligible for analysis. Upon passing QC, GFP and CD69 (T cells) and αHA (B cell labeling) are used to determine TCR activation signal strengths and call confidence. (**B**) Representative flow cytometry data for two TCRs (P20, upper panels; P2, lower panels) co-cultured with class II HLA-deficient autologous B-LCLs. Gray histograms, control peptide-loaded APCs; green histograms, cognate peptide-

loaded APCs. TCR specificity for the cognate epitope in complex with the restricted HLA allele is lost upon deficiency of the respective allele (e.g. HLA-DR for P20, HLA-DQ for P2). (**C**) Patient ID07 class II HLA haplotype.

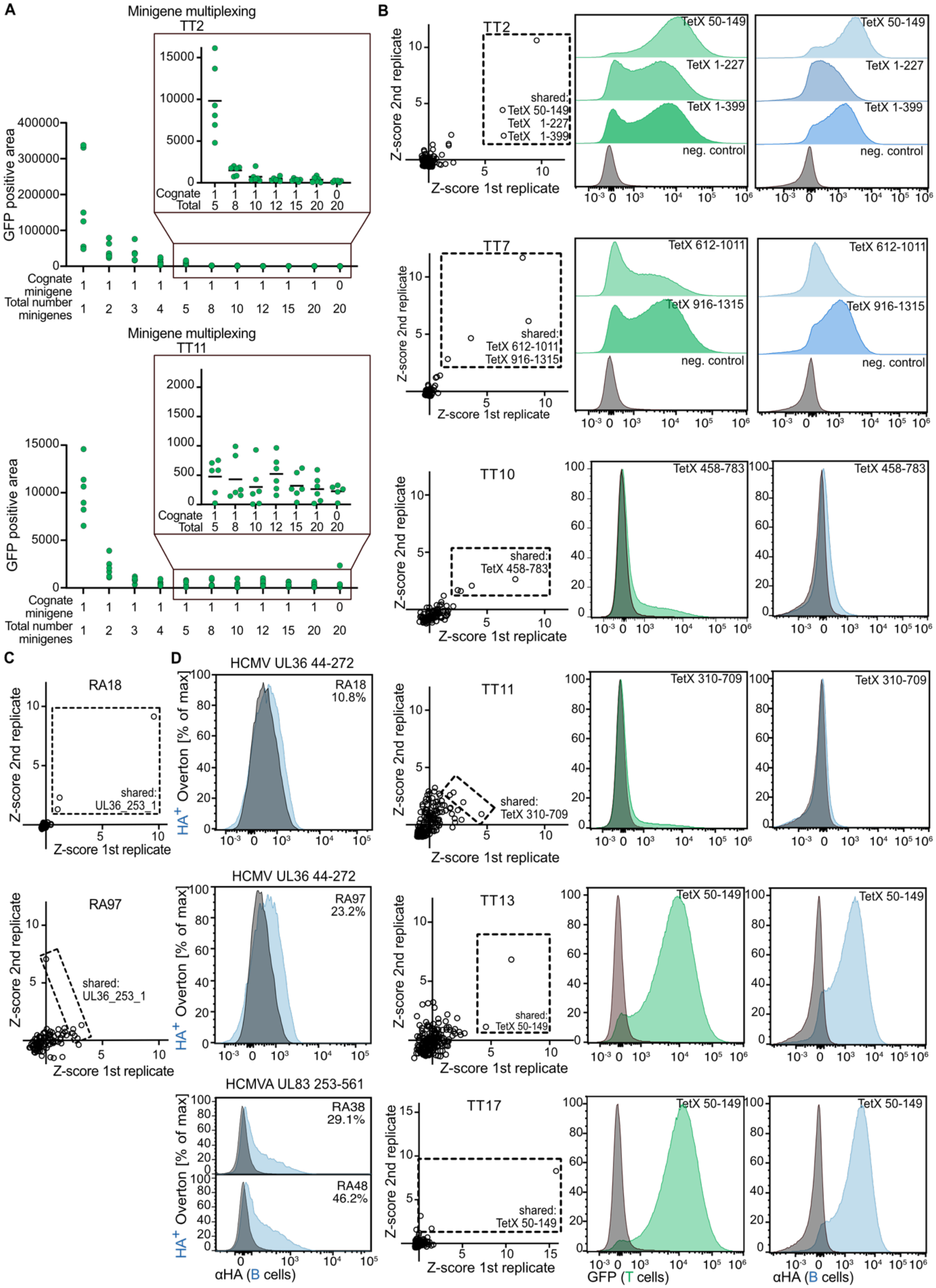

A
Minigene multiplexing
TT2
GFP positive area
Cognate minigene
Total number minigenes
Minigene multiplexing
TT11
B
TT2
Z-score 2nd replicate
Z-score 1st replicate
shared:
TetX 50-149
TetX 1-227
TetX 1-399
neg. control
TT7
shared:
TetX 612-1011
TetX 916-1315
TT10
shared:
TetX 458-783
C
RA18
shared:
UL36_253_1
RA97
D
HCMV UL36 44-272
RA18
10.8%
RA97
23.2%
HA+ Overton [% of max]
TT11
shared:
TetX 310-709
TT13
shared:
TetX 50-149
HCMVA UL83 253-561
RA38
29.1%
RA48
46.2%
αHA (B cells)
TT17
GFP (T cells)
αHA (B cells)

Fig. S4. TCR ligand identification from putative target libraries. (**A**) Putative target multiplexing. An increasing number of non-cognate targets was added at equimolar ratios to the mixture of minigenes used to transduce B-LCLs prior to co-culture with reporter T cells bearing TCRs (the strongly reactive TT2 or the weakly reactive TT11) specific for a single target in the mixture. (**B**) Plate-based antigen discovery for six tetanus toxoid-reactive TCRs. Identification of positive wells with shared minigenes (dashed boxes) in an array containing 162 putative targets across 96 wells. The screen was performed in duplicate (x and y axes) to better identify reproducibly positive wells. Histograms to the right: reporter T cell activation in single-target minigene co-culture for the respective arrayed screen results on the left. (**C**) Plate-based antigen discovery for two and (**D**) single-target validation of four rheumatoid arthritis TCRs.

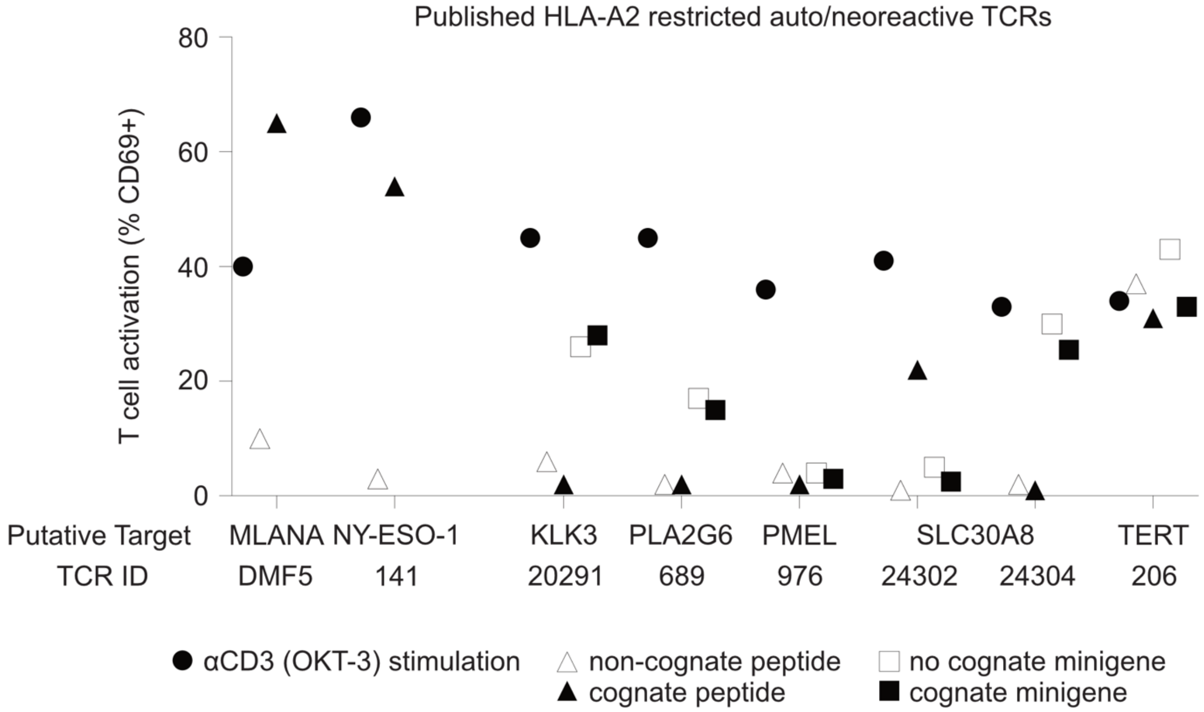

Fig. S5. Public-domain TCRs do not functionally activate T cells in response to published putative targets. TCRs with HLA-A2 restriction and human protein epitope targets were collected from online repositories and tested in co-culture with target-containing minigenes or 1 µM loaded peptide. TCR surface expression and ligand-independent functionality were assessed by αCD3 stimulation (black circles). DMF5 and NY-ESO-1_141 are included as positive controls; minigene data for these TCRs is presented in main Fig. 3A.
